# Supplementary material for: Comparative Personality Traits Assessment of Three Species of Communally Housed Captive Penguins
Source: Animals (Basel). 2019 Jun 20;9(6):376. doi: 10.3390/ani9060376 (PMC6616866; doi:10.3390/ani9060376)
Supplement: Supplementary file 1 [file animals-09-00376-s001.pdf]

animals

MDPI

Supplementary Files: Comparative Personality Traits Assessment of Three Species of Communally Housed Captive Penguins

Giovanni Quintavalle Pastorino [1], Richard Preziosi [2], Massimo Faustini [1], Giulio Curone [1], Mariangela Albertini [1], Dawn Nicoll [3], Lorna Moffat [3], Romain Pizzi [3] and Silvia Mazzola [1,*]

Table S1. Animals involved in the study, K as king penguin (*Aptenodytes patagonicus*), NR as northren rockhopper (*Eudyptes moseley*) and G as gentoo penguin (*Pygoscelis papua*).

Identification Code	Penguin Name	Band Color/Side	Species	Date of Birth	Gender	Rearing History
88HB7/27,655,951	Blue	Blue	K	08/30/1988	Male	Parent reared
B04C01/10.443,092	Nils	Purple R	K	07/30/2002	Male	Hand reared
B04H07/27,655,863	Fingal	White R	K	08/15/2004	Male	Parent reared
B09J02/27,701,744	Maclean	Red/Yellow R	K	10/22/2009	Male	Unknown
B13K01/BQK1200636	Bow	Red/Green R	K	09/14/2012	Male	Parent reared
B14K02/25,462,691	Yoepie	Gold R	K	09/22/1997	Male	Parent reared
B16L01/QQT1501700	Alfie	Lt, Blue/Green R	K	07/18/2015	Male	Parent reared
B17B01/BQK15-1257	Kongo	Orange R	K	08/28/2015	Male	Unknown
B15J03/QQT1401498	Dennis	Black/Red R	K	07/17/2014	Male	Unknown
870514/27655853	Mrs Wolowitz	Ora/White L	NR	05/13/1987	Female	Parent reared
89EB9/27,655,851	Mrs White	White L	NR	03/05/1989	Female	Parent reared
91EB03/17,397,775	Helena	Dk, Blue/White L	NR	05/05/1991	Female	Parent reared
B06J03/27655870	Dwaine	Pink/Green R	NR	04/29/1988	Male	Parent reared
B06J07/27655871	Nestor	Dk, Blue/Pink R	NR	08/04/1993	Male	Parent reared
B08D03/27,655,862	Tristan	Lt, Blue/Yell R	NR	04/26/2008	Male	Parent reared
B09D08/MIG1228158516	Eddie	Black/Green R	NR	04/27/2009	Male	Parent reared
B09D12/MIG1228158533	Issy	Lt, Blue/Yellow L	NR	04/29/2009	Female	Parent reared
B12B01/MIG1230087409	Gordon	Brown/Gold R	NR	09/04/2008	Male	Unknown
B12B02/MIG1230087410	Isla	Gold/Pink L	NR	04/14/2009	Female	Hand reared
B12B03/26,295,973	Millie	Gold/Yellow L	NR	04/16/2009	Female	Hand reared
B12B04/ZBQ12-01319	Al	Gold/Orange R	NR	01/05/2010	Male	Hand reared
B12B05/ZBQ1201320	Balboa	Gold/Pink R	NR	04/23/2010	Male	Parent reared
B12B06/ZBQ12-01321	Jura	Gold/Yellow R	NR	04/21/2010	Male	Parent reared
B12B07/ZBQ12-01322	Wesley	Gold/White R	NR	04/28/2010	Male	Parent reared
B12B08/ZBQ12-01323	Bruce	Dk Blue/Gold R	NR	04/21/2011	Male	Parent reared
B12B09/ZBQ12-01324	Penny	Brown/Gold L	NR	04/21/2011	Female	Parent reared
B12B10/ZBQ12-01325	Pinhead	Gold/Orange L	NR	04/16/2011	Female	Parent reared
B12B11/ZBQ12-01326	Amy	Gold/White L	NR	04/24/2011	Female	Parent reared
B12B12/ZBQ12-01327	Brucetta	Dk, Blue/Gold L	NR	04/25/2011	Female	Parent reared
B17D01/ZBQ17-06348	Batman	Gold R	NR	04/25/2017	Male	Parent reared
90EB77/26,975,204	Boy	Blue/Ora/Yell R	G	05/20/1990	Male	Parent reared
91EB39/26,774,871	Mrs Spain	Orange/White L	G	05/19/1991	Female	Parent reared
94EB32/25,263,018	BB	Dk, Blue/Lt,Blue L	G	01/05/1994	Female	Parent reared
96DB11/22,765,004	Snowflake	Unbanded	G	04/30/1996	Male	Parent reared
97EB33/2,744,168	Mary	Dk, Blue/Red L	G	12/05/1997	Female	Parent reared
98DB10/26,975,197	Mrs Colin	Lt, Blue/Grey L	G	04/28/1998	Female	Parent reared
98EB19/26,975,193	Colin	Dk, Blue/Yellow R	G	03/05/1998	Male	Parent reared
B00E18/MIG1228158458	Boo	Lt, Blue/White L	G	05/27/2000	Female	Parent reared
B01E01/26,774,831	Poppet	Dk blue/White L	G	12/05/2001	Female	Parent reared
B02E07/26774894	Dolores	Grey/white L	G	05/16/2002	Female	Parent reared
B03E07/26,975,202	Buzz	Lt, blue/grey R	G	12/05/2003	Male	Parent reared
B04E05/26,774,874	Mr Spain	Red/yellow R	G	05/15/2004	Male	Parent reared
B16D02/JFQ15-07182	Chip	Orange/yellow R	G	07/07/2015	Male	Unknown
B16E11/ZBQ16-06160	Kevin	Gold R	G	12/05/2016	Male	Hand reared

Table S2. Personality traits included in the questionnaire, that had been rated by the keepers.

Personality Trait	Description of the Trait Characteristic
Active	Moves around enclosure (e.g., walks, runs)
Aggressive to conspecifics	Reacts hostile (e.g., attacks) toward other penguins
Aggressive to familiar people	Reacts hostile and threatening towards familiar people and staff members
Aggressive to keepers	Reacts hostile and threatening towards keepers
Aggressive to unfamiliar people	Reacts hostile and threatening towards unfamiliar staff and members of the public
Aggressive to you	Reacts hostile and threatening towards you
Calm	Not easily disturbed by changes in the environment
Cooperative	Is compliant; willingly behaves when asked to do something (e.g., during training)
Curious	Approaches and explores changes in the environment (e.g., enriching and novel objects)
Dominant	Dominant to conspecifics: in a commanding or elevated position compared to conspecifics
Eccentric	Shows stereotypic or unusual behaviors
Excitable	Overreacts to changes in the environment
Friendly to conspecifics	Initiates and seems to seek proximity to other penguins
Friendly to keepers	Initiates proximity with keepers; approaches barriers readily and in a friendly manner
Friendly to familiar people	Initiates proximity with familiar visitors; approaches fence readily and in a friendly manner
Friendly to unfamiliar people	Initiates proximity with unfamiliar visitors; approaches fence readily and in a friendly manner
Friendly to you	Initiates proximity with you; approaches fence readily and in a friendly manner
Fearful of conspecifics	Retreats and hides from other penguins
Fearful of familiar people	Retreats and hides from familiar people and staff members
Fearful of unfamiliar people	Retreats and hides from unfamiliar staff and members of the public
Fearful of keepers	Retreats and hides from keepers
Fearful of you	Retreats and hides from you
Insecure	Seems scared easily; "jumpy" and fearful in general
Playful	Initiates and engages in play behavior (seemingly meaningless, non-aggressive behavior) with objects and/or other penguins
Self-assured	Moves in a seemingly confident, well-coordinated and relaxed manner
Smart	Learn quickly to associate certain events and appears to remember for a long time
Solitary	Spends time alone; avoids company
Tense	Shows restraint in movement and posture
Timid/shy	Reluctance to approach other animals, novel objects, or new situations
Vocal: aggressive	Frequently and readily vocalizes aggressively
Vocal: non-aggressive	Frequently and readily vocalizes (friendly or neutral vocalizations)

Table S3. Kruskal-Wallis non-parametric test analysis of the differences between the median values of the personality traits rated by the keepers in the three species of penguin. Only statistically different results are listed, χ^2 = chi-square values; df = degree of freedom values, $p < 0.05$ values are considered as statistically different.

Kruskal-Wallis			
	χ^2	df	*p*
Active	7884	2	0.019
Calm	8462	2	0.015
Curious	5903	2	0.052
Friendly to other penguins	8335	2	0.015
Playful	9696	2	0.008
Vocal: aggressive	9463	2	0.009
